# Biomarkers of Gamma-Hydroxybutyric Acid (GHB) Exposure: A Comprehensive Review of Analytical and Forensic Advances

**DOI:** 10.3390/toxics13100824

**Published:** 2025-09-27

**Authors:** Alice Voisin, Caroline Solas-Chesneau, Anne-Laure Pélissier-Alicot, Nicolas Fabresse

**Affiliations:** 1 Laboratory of Pharmacokinetics and Toxicology, La Timone University Hospital, 264 Rue Saint Pierre, 13385 Marseille Cedex 5, France; alice.voisin@ap-hm.fr (A.V.); caroline.solas@ap-hm.fr (C.S.-C.); 2 Legal Medicine Department, La Timone University Hospital, 13005 Marseille, France; anne-laure.pelissier@ap-hm.fr; 3 SESSTIM, Economic and Social Sciences of Health and Medical Information Processing, INSERM, Aix Marseille University, 13005 Marseille, France

**Keywords:** GHB, biomarkers, forensic toxicology, mass spectrometry, detection window

## Abstract

Gamma-hydroxybutyric acid (GHB) is a short-chain fatty acid with both endogenous and exogenous origins, complicating its detection in clinical and forensic toxicology. Due to its rapid metabolism and short detection window in conventional biological matrices, identifying reliable biomarkers of GHB exposure is crucial. This literature review aims to assess current knowledge on potential GHB biomarkers that may extend the detection window or improve specificity. A systematic search of scientific databases was conducted to identify studies investigating GHB metabolites, conjugates, and related biochemical markers using advanced analytical techniques such as LC-MS/MS and GC-MS. The review highlights promising candidates, including glycolic acid, carnitin-GHB, and glycin-GHB, as well as 3,4-dihydroxybutyric acid, which show potential for distinguishing exogenous intake. However, significant interindividual variability and limited validation studies hinder their widespread implementation. Despite promising findings, further research is needed to confirm the specificity, stability, and reproducibility of these biomarkers. This review underscores the importance of developing standardized protocols to enhance GHB exposure detection in both clinical and forensic settings.

## 1. Introduction

Gamma-hydroxybutyric acid (GHB) is a naturally occurring short-chain fatty acid synthesized from gamma-aminobutyric acid (GABA), a key neurotransmitter in the central nervous system. Although GHB is endogenously present in the human body, it is also used exogenously for therapeutic purposes (narcolepsy, anesthesia), and is known for its illicit use in recreational and criminal contexts [1]. Due to its euphoric, sedative, and disinhibitory effects, GHB is commonly misused during “chemsex” practices, particularly among men who have sex with men, and has also been implicated in drug-facilitated sexual assaults [2,3]. These illicit uses, combined with its narrow therapeutic index and rapid metabolism, make GHB a challenging substance to monitor and regulate in both clinical and forensic settings.

One of the main limitations in detecting GHB intake lies in its endogenous production and rapid elimination, resulting in a very short detection window—typically less than 6 h in blood and 12 h in urine [4]. Moreover, post-mortem formation and the variability in baseline endogenous levels complicate the interpretation of toxicological results [5]. These challenges have sparked a growing interest in identifying specific biomarkers that could widen the detection window and provide more reliable evidence of exposure to exogenous GHB.

The purpose of this work is to conduct a comprehensive review of the literature to identify and evaluate biomarkers that could serve as reliable indicators of GHB exposure. This review synthesizes current knowledge, highlights analytical strategies, and discusses the strengths and limitations of various biomarkers and matrices. The ultimate aim is to improve detection capabilities in both clinical and medico-legal toxicology, contributing to more accurate diagnosis, forensic investigation, and public health interventions in the context of GHB misuse.

## 2. Materials and Methods

This literature review was conducted using a structured approach to identify and analyze relevant publications on biomarkers of exposure to gamma-hydroxybutyric acid (GHB). Scientific articles were retrieved from the PubMed/MEDLINE and Science Direct databases. The search strategy included the following keywords, used alone and in combination with Boolean operators: “*GHB*” *OR* “*gamma-hydroxybutyrate*” *OR* “*gamma-hydroxybutyric acid*” AND “*biomarker*” *OR* “*metabolites*” *OR* “*amino acids*” *OR* “*carnitines*” *OR* “*conjugates*”. Only original research articles published in English and directly focused on the identification, characterization, or evaluation of biomarkers associated with GHB exposure were included. No date restriction was applied, but preference was given to the most recent and methodologically robust publications. There were no restrictions on the type of study. This query returned 264 articles on PubMed; 242 articles were not directly related to the subject and were excluded. After exclusion of reviews (n = 2), a technical study of the synthesis of biomarkers (n = 1) and studies not directly relevant to biomarker development in blood or urine, particularly hair (n = 2), nails (n = 1) and organs (n = 1), 15 papers were selected and analyzed. An additional paper was found in Microchemical Journal, a journal that is only indexed by ScienceDirect [6]. An article cited by four of the studies included in this review has been added; this one was published in a journal that was not indexed at the time of publication (Metabolomics, now indexed in PubMed/MEDLINE).

Priority was given to studies using validated analytical techniques such as gas chromatography–mass spectrometry (GC-MS), liquid chromatography–tandem mass spectrometry (LC-MS/MS), and high-resolution mass spectrometry (LC-HRMS). Data extracted included the studied population, biological matrix, analytical technique, biomarker type, and performance characteristics (e.g., sensitivity, specificity, detection window). No animal or human experimentation was performed as part of this review.

## 3. Results and Discussion

The detection of exogenous gamma-hydroxybutyric acid (GHB) poses significant challenges in both clinical and forensic toxicology. These difficulties are primarily due to the compound’s dual endogenous and exogenous origin, its rapid metabolism, and its narrow detection window in conventional biological matrices such as blood and urine. In routine practice, the ability to discriminate between physiological and illicit GHB concentrations remains limited because the concentrations overlap. The present literature review offers a comprehensive overview of the biomarkers that have been proposed to address these analytical and interpretive challenges. The main information contained in the included studies is summarized in Table 1. A summary of the sample preparation and analytical techniques used in each of the studies included in the review is provided in Table S1.

Study design can be grouped into two categories: hypothesis-driven approaches and metabolomics studies. Hypothesis-driven studies can be further divided into two groups: studies focusing on GHB metabolites by analogy with ethanol metabolism (e.g., GHB glucuronide, GHB sulfate, and phosphatidyl GHB), and studies focusing on indirect markers reflecting GHB-induced metabolic alterations (e.g., organic acids, amino acids, and polyamines).

Petersen demonstrated the presence of GHB-glucuronide in GHB-negative urine samples (GHB < 0.5 µg/mL) [10]. Subsequently, Hanish et al. identified GHB sulfate in the urine of all GHB-positive patients (GHB: 70–170 µg/mL) and in 20% of GHB-negative samples. Following these publications, Piper et al. evaluated the potential of these biomarkers to extend the GHB detection window to 72 h by conducting a study involving the controlled administration of GHB [12]. However, these two markers were not detectable after 72 h. Subsequently, Steuer et al. evaluated these two biomarkers in a placebo-controlled crossover study; however, no significant difference was observed between users and non-users, highlighting their possible endogenous origin or rapid conjugate clearance [14]. These findings suggest that, despite their theoretical interest, these GHB conjugates may lack the specificity necessary for confident attribution of exogenous intake in forensic contexts. Studies are now turning to metabolites formed by phospholipid esterification, by analogy with phosphatidylethanol, which extends the detection window of ethanol [19]. However, to date, no studies have been conducted in real subjects to assess the relevance of these biomarkers.

In parallel, attention has shifted to metabolic by-products, particularly organic acids, polyamines, and amino acid derivatives. Experimental studies in animals (e.g., [7,8]) and controlled studies in humans (e.g., [16]) have revealed changes in metabolites such as glycolic acid, 3,4-dihydroxybutyric acid, and 2-hydroxyglutaric acid after GHB administration. These findings suggest that exogenous GHB induces measurable perturbations in several metabolic pathways, particularly those related to GABA metabolism and the tricarboxylic acid cycle. By analyzing 472 urine samples from 206 healthy women, Kim et al. sought to define reference concentrations for these metabolites and identify factors of variability [20]. However, the specificity of these metabolites is still a matter of debate, given that their concentrations can be influenced by physiological factors, diet, or other exogenous substances. Furthermore, significant inter-individual variability makes it difficult to establish reliable thresholds for interpretation.

Recent advances in high-resolution metabolomics have enabled the identification of novel GHB-related metabolites, such as amino acid conjugates (GHB-glycine, GHB-taurine), GHB-carnitine, GHB-pentose, or 4-guanidinobutyric acid (GBA). Studies by Steuer et al. (2021) and Wang et al. (2022) report that some of these compounds show strong discriminatory potential between exposed and non-exposed individuals, particularly when analyzed through multivariate statistical models [15,18]. Subsequently, Steuer et al. synthesized some of these biomarkers and validated methods for quantifying them in order to assess their usefulness in cohorts of patients treated with GHB [22,23]. GHB-glycine was the most promising of these biomarkers, enabling the detection window to be extended to 28 h. Two interesting biomarkers (M259T82 and M507T82) that were highlighted in previous studies were not investigated here due to difficulties in identifying them. To date, GBA has only been identified in rat urine. Human studies should be conducted to confirm its significance. These emerging biomarkers represent a promising avenue, especially when used in combination, though they remain in early stages of validation. Standardization across analytical platforms and larger, diverse study cohorts will be essential to confirm their diagnostic value and applicability in routine toxicological investigations.

In terms of matrices, urine remains the most commonly studied due to its ease of collection and the longer excretion time for certain metabolites. Nevertheless, matrices such as serum, whole blood, vitreous humor, and hair are also of interest. To date, hair is the only matrix that allows for an extended detection window [24]. However, hair analysis is difficult to implement; it is only carried out by a few specialized laboratories and interpretation is complex. This makes it hard to implement systematically in cases of suspected GHB poisoning. Serum and whole blood offer narrower windows but may better reflect acute intoxication, especially blood in postmortem cases. Vitreous humor seems to be less affected than peripheral blood by GHB postmortem production, and several cut-off concentrations have been proposed to distinguish endogenous production of GHB in vitreous humor from antemortem ingestion of GHB [25]. However, in GHB-related fatalities, vitreous humor is rarely used for GHB quantitation. Combining multiple matrices may ultimately provide a more complete toxicological picture.

Collectively, these findings emphasize the need for a multi-marker and multi-matrix approach. The sole reliance on free GHB measurement is no longer sufficient in many forensic and clinical scenarios. However, despite significant progress, no single biomarker currently fulfills the criteria of specificity, sensitivity, and robustness required for conclusive determination of GHB exposure. Integrating metabolomics, lipidomics, and proteomics approaches could be a transformative development in gamma-hydroxybutyric acid (GHB) research. This can offer new ways to identify biomarkers and improve our understanding of GHB metabolism. Integrated multi-omics approaches may offer a holistic view of biological responses to GHB exposure. This multidimensional approach enables researchers to construct comprehensive molecular networks illustrating the interconnected biological processes affected by GHB, from initial absorption through to metabolic clearance. Applying these omics methodologies could significantly improve forensic toxicology practices by identifying stable metabolic markers that remain detectable long after GHB has been eliminated from the body.

The heterogeneity in study design, analytical protocols, and sample handling across the available literature complicates the interpretation and comparison of findings. Future research should prioritize standardized protocols, including pre-analytical handling, normalization techniques, and interpretation frameworks. Inter-laboratory collaborations and shared databases could accelerate biomarker validation and consensus-building. In addition, exploration of pharmacogenomic factors that influence GHB metabolism may reveal interindividual susceptibilities and contribute to personalized toxicological assessments.

## 4. Conclusions

The detection of exogenous gamma-hydroxybutyric acid (GHB) remains a significant challenge due to its endogenous presence, rapid metabolism, and short detection window. This review highlights the breadth of research aimed at identifying reliable biomarkers capable of improving GHB exposure assessment. While organic acids and novel derivatives like GHB-glycine offer promising leads, none currently provide definitive proof of exogenous intake when used alone. The literature emphasizes the need for multi-marker strategies, validation in large and diverse populations, and methodological standardization. Future work should also explore alternative matrices and metabolomic profiling to improve both sensitivity and specificity. Ultimately, the integration of emerging biomarkers into routine toxicological practice could enhance the accuracy and robustness of GHB exposure investigations in both clinical and forensic settings.

## Figures and Tables

**Table 1:** The main information relating to the studies included in the review is presented, including their objectives, the populations studied, and the key findings.

Reference	Objectives	Populations Studied (n)	Matrix	Key Findings
Animal study (Sprague-Dawley rats)				
Seo et al., 2016 [7]	Study the profile of organic acids (OAs), including those bound to the tricarboxylic acid (TCA) cycle, as well as GHB and 2-hydroxyglutaric acid (2-HG), in rat urine samples following the intraperitoneal injection of GHB.	Control group (n = 6): urine collected before GHB injection. Group 1 (n = 6): single intraperitoneal administration (2.6 g/kg), urine was collected on the 2nd day. Group 2 (n = 6): multiple intraperitoneal administrations, urine collected on the 11th day. after first administration (2.6 g/kg/day). Six animals were used in the experimental part, each animal being its own control.	Urine	In group 1 and 2, ketoglutaric acid was the most abundant, followed by citric and isocitric acids among the 18 AOs quantified (LOD = 0.01–55.4 ng/mL). An analysis of variance test revealed a significant difference between the control group and the other two groups in 15 AOs (*p <* 0.05*)*: 2-hydroxybutyric acid, 2-hydroxyglutaric acid, acetoacetic acid, alpha-ketoglutaric acid, c*is*-aconitic acid, citric acid, fumaric acid, glycolic acid, isocitric acid, lactic acid, malic acid, malonic acid, oxaloacetic acid, pyruvic acid, succinic acid. The metabolic disruption of AOs after single administration is greater than after multiple administration.
Seo et al., 2018 [8]	Determine the urinary amino acids (AA) profile in rat urine samples after intraperitoneal injection of GHB.	N = 25 Injection of 600 mg/kg GHB Control group: urine collected before injection. Group 1: single administration, collection 12 h after single administration. Group 2: multiple administrations, collection 10 days after daily administration.	Urine	Among a total of 28 AA analyzed (limit of detection from 0.01 to 8.47 ng and limit of quantification from 0.01 to 14.51 ng), significant differences were observed in the levels of 26 AA between the control, single and multiple administration groups: α-aminoadipic acid, α-aminobutyric acid, alanine, aspartic acid, asparagine, β-aminoisobutyric acid, glycine, glutamic acid, glutamine, homocysteine, histidine, isoleucine, lysine, leucine, methionine, N-methyl-DL-aspartic acid, ornithine, phenylalanine, pipecolic acid, proline, pyroglutamic acid, serine, threonine, tryptophan, tyrosine, valine, γ-aminobutyric acid, 4-hydroxyproline. Levels of GABA were significantly different between the control group compared with the single administration group, but not with the multiple administration group.
Lee et al., 2019 [9]	Determine the urinary polyamines (PAs) profile in rat urine samples after intraperitoneal injection of GHB.	N = 6 Injection of 600 mg/kg GHB Control group: urine collected before injection. Group 1: single administration, collection at the second day after single administration. Group 2: multiple administrations, collection 11 days after daily administration.	Urine	There were significant differences in the levels of N^1^-acetylspermine, putrescine, N^1^-acetylspermidine, and spermine between the control group and group 1 (*p* = 0.001, *p* = 0.006, *p* = 0.009 and *p* = 0.027, respectively). In the group receiving multiple administrations, the levels of N^1^-acetylspermine and spermine increased significantly, by 171% and 70%, respectively, compared to the control group. Meanwhile, the levels of N^1^-acetylspermidine and putrescine decreased significantly, by 35% and 22%, respectively. N^1^-acetylspermine was identified as the main polyamine that could be used to discriminate between the control group and the single-dose and multiple-dose GHB groups. Similar levels of spermine were observed in the single-dose and multiple-dose groups. Combined with N^1^-acetylspermine, this makes spermine a potential biomarker for GHB exposure and dependence.
Kim et al., 2025 [6]	Identify new biomarkers of gamma-hydroxybutyrate (GHB) intoxication by analyzing urinary metabolic alterations in rats with a targeted and untargeted metabolomic approach	N = 20 Injection of 600 mg/kg GHB For both targeted and non-targeted metabolomic analyses: Control group: N = 6 Test group: N = 5 Urine was collected over 12 h. For assessing the GHB detection window: Control group: N = 5 Test group: N = 4 Urine samples were collected every 3 h for 24 h.	Urine	Targeted metabolomics: Urinary concentrations of GHB and its metabolites (GABA (LOD = 0.01 µg/mL), glutamic acid (LOD = 0.06 µg/mL), succinic acid (LOD = 0.3 µg/mL), 2,4-OH-BA (LOD = 0.24 µg/mL), 3,4-OH-BA (LOD = 0.21 µg/mL), and glycolic acid (LOD = 3 µg/mL)) were significantly increased following a 600 mg/kg GHB administration in rats over a 12-h period. Creatinine-adjusted concentrations showed substantial fold changes in the GHB group compared to controls: 2,4-OH-BA (16.2-fold), glutamic acid (13.9-fold), 3,4-OH-BA (12.0-fold), GABA (11.7-fold), succinic acid (6.5-fold), and glycolic acid (2.8-fold). 4-Guanidinobutyric acid (GBA) was identified as a novel biomarker for GHB intoxication. Urinary GBA levels peaked at 3 h post-administration before declining, following a similar pattern to GHB and other metabolites. GBA levels in the GHB group were significantly higher than controls, with creatinine-adjusted concentrations of 73.0 ± 16.2 µg/mg creatinine at 9 h, compared to 39.0 ± 11.2 µg/mg creatinine in the control group (*p* < 0.01). Untargeted metabolomics: Significantly elevated metabolites in the GHB group, including GBA, xanthurenic acid, riboflavin, pyridoxine, indole-3-acetate, hippurate, thymidine, N-acetyl-L-leucine, azelaic acid, 4-pyridoxate, 4-hydroxybenzoate, 4-coumarate, and GHB, were identified and confirmed by MS/MS spectral matching.
Human study				
Petersen et al., 2013 [10]	Detect GHB-glucuronide in human urine samples (GHB-GLU).	N = 50 Samples were drawn from healthy volunteers.	Urine	GHB was below the LOQ (<0.5 μg/mL) in all samples (n = 50) (LOD = 0.025 mg/mL). GHB-GLU was detected in all samples, mean concentration = 1.3 µg/mL (n = 47), three samples were below the limit of quantification (0.5 µg/mL).
Hanish et al., 2015 [11]	Detect GHB-sulfate (GHB-SUL) in urine samples from patients using GHB or its precursors.	N1: GHB-positive urines between 70 and 170 μg/mL (n = 5). N2: GHB-negative urines (n = 5).	Urine	N2: GHB-SUL not detected in 4/5 samples. N1: GHB-SUL detected concomitantly with GHB in 5/5 samples. No quantification of GHB-SUL was performed in this study.
Piper et al., 2017 [12]	Evaluation of GHB-SUL and GHB-GLU metabolites in different populations to extend the GHB detection window.	N1 = 100 male and female athlete urine samples. N2 = 50 urine samples from students. N3 = 3 volunteers’ urine after administration of 1.86 g of GHB orally; urine was collected 72 h later.	Urine	No significative difference was found between N1 and N2 for GHB-GLU and GHB-SUL (*p* < 0.05) (LODs < 10 ng/mL covering a linear range up to 1 mg/mL (GHB_SULF) and 20 mg/mL (GHB_GLUC)). There was no significant difference between the male and female populations, and no difference between athletes and non-athletes. There was no significant difference between N3 and N1 or N2. These two biomarkers are not suitable for extending the detection window.
Palomino-Schätzlein et al., 2017 [13]	Suitability of NMR spectroscopy for the monitoring of exogenous GHB in body fluids, and interest in metabolomics.	N = 12 (6 male/6 female). Single dose of 25 mg/kg GHB administered orally. Urine collected at T10min, T1h, T2h, T4h, T6h, T14h, T20h, T24h and T30h. Blood collection at T10min, T1h and T13h.	Urine and serum	This study confirmed the feasibility of NMR spectroscopy for measuring exogenous GHB in body fluids such as urine and serum, as well as the value of the NMR-based metabolomics approach for biomarker research. The study highlighted a significant increase in the concentration of glycolate and succinate in urine after GHB ingestion. Glycolate present a longer detection window compared to GHB and succinate.
Steuer et al., 2019 [14]	To identify possible new biomarkers of GHB consumption by evaluating urine samples obtained in a randomized, placebo-controlled crossover study. N = 20 male volunteers received 50 mg/kg sodium oxybate (Xyrem^®^) at night, in the middle of a sleep episode.	N1 = 10 healthy volunteers, GHB administration, 42 mg/kg orally. N2 = 20 placebo healthy volunteers, placebo administration. All samples were collected at T4.5h.	Urine	GHB significantly increased in N1 (*p <* 0.05). GHB-GLU and GHB-SUL were detected in N1 and N2, but no significant difference was observed between the two groups. GHB-carnitine, GHB-glutamate, and GHB-glycine were present in all N1 samples. Glycolic acid and succinylcarnitine were present in N1 and N2 but significantly increased in N1. GHB can be catabolized to acetyl-CoA and glycolate by β-oxidation and converted to 3-hydroxypropionyl-CoA by α-oxidation.
Steuer et al., 2021 [15]	Identify new biomarkers of GHB using metabolic profiling in serum and urine samples collected in a placebo-controlled crossover study in healthy men.	N1 = 10 GHB administration, 42 mg/kg orally. Serum collected at T0, T4, and T16.5h. Urine at T4.5h and T8h. N2 = 10 (placebo) Serum collected at T0, T4, and T16.5h. Urine at T4.5h and T8h. N3 = 15 (administration of 42 mg/kg GHB orally Urine collected at T8h.	Urine and serum	Urine T4.5h: significant increase in GHB-glutamate, GHB-glycine, GHB-taurine, GHB-carnitine, succinylcarnitine, glycolic acid conjugate with the amino acid taurine. Urine T8h: decrease in all compounds. The results suggest that GHB-pentose and a glycolic acid conjugate are the most promising metabolites. Serum: only dihydroxybutyric acid and 2-methylbutyroxylcarnitine appear to be affected by GHB intake. With the exception of traces of GHB-glycine, none of the above-mentioned urinary biomarkers could be detected. The concentration of unidentified compounds (M259T82, M507T82) decreases very little between the T4.5h and T8h urine samples.
Jarsiah et al., 2021 [16]	Evaluation of glycolic acid, succinic acid, 3,4-dihydroxybutyric acid, and 2,4-dihydroxybutyric acid as biomarkers to extend the GHB window detection.	N = 17 patients positive for GHB (10 serum samples and 7 urine samples). Threshold values: >4 µg /mL in serum or > 10 µg/mL in urine. N = 40 alleged cases of drug-facilitated sexual assault (DFSA) with negative GHB results (21 serum and 19 urine samples).	Urine and serum	In all serum samples positive for GHB, glycolic acid (GA), 3,4-dihydroxybutyric acid (3,4-OH-BA), and 2,4-dihydroxybutyric acid (2,4-OH-BA) were higher in comparison with the population of alleged cases of DFSA. 5/7 GHB positive urine samples presented increased concentrations of GA, 3,4-OH-BA, and 2,4-OH-BA. 2/7 did not present an increase, this was associated to GHB concentrations close to the positivity threshold (>10 µg/mL). Among GHB-negative serum samples, 6 showed increased concentrations of GA, 3,4-OH-BA, and 2,4-OH-BA suggesting an intake of GHB or its precursors. Serum analysis is more reliable than urine analysis. Thresholds were recommended for interpreting potential cases of ante mortem GHB intoxication: - 3,4-OH-BA: >3 µg/mL in serum and >50 µg/mL in urine - 2-OH-BA: >2 µg/mL in serum and >25 µg/mL in urine - GA: >5 µg/mL in serum and >400 µg/mL in urine In this study, the limits of detection were 0.12 mg/L for 2,4-OH-BA butyric acid, 0.13 mg/L for 3,4-OH-BA, 0.03 mg/L for GHB, 0.28 mg/L for SA, and 0.19 mg/L for GA, respectively. Limits of quantification were 0.39 mg/L for 2,4-OH-BA, 0.42 mg/L for 3,4-OH-BA, 0.11 mg/L for GHB, 0.98 mg/L for SA, and 0.63 mg/L for GA.
Küting et al., 2021 [17]	Detect GHB administration via detection elevated concentrations of 6 organic acids (adipic acid (AA), 4-hydroxyphenylacetic acid (4-HPA), glycolic acid (GA), succinic acid (SA), 3,4-dihydroxybutyric acid (3,4-DHB), and 2,4 dihydroxybutyric acid (2,4-DHB) in plasma and urine.	N = 5 samples were collected from narcolepsy patients treated with either Xyrem^®^ (Patients 1, 2, 4, 5) or Somsanit^®^ (Patient 3).	Plasma and urine	Concentration curves for adipic acid (AA), 4-hydroxyphenylacetic acid (4-HPA), glycolic acid (GA), and succinic acid (SA) in plasma showed no clear increase or decrease within 12 h of GHB ingestion. Similar results were observed for AA, 4-HPA, and SA in urine. Following the administration of GHB, the concentrations of 3,4-DHB and 2,4-DHB in plasma increased. However, the increase in 2,4-DHB concentration was much lower than that in 3,4-DHB. Following GHB administration, an increase in the concentration of 3,4-DHB, 2,4-DHB and GA was observed in urine. Baseline concentrations (standardized to creatinine) were found at: - GA: 6.5–22 h after ingestion - 3,4-DHB: 11.5–22 h - 2,4-DHB: 8.5–70 h. In this study, the LODs were: GA = 0.23 μg/mL; GHB = 0.05 μg/mL; SA = 0.08 μg/mL; 2,4-DHB = 0.07 μg/mL; 3,4-DHB = 0.06 μg/mL; AA = 0.07 μg/mL; 4-HPAA = 0.07 μg/mL.
Wang et al., 2022 [18]	Investigate a range of advanced analytical methods to discover those best suited to detect new and known direct biomarkers/adducts of GHB consumption in routine UHPLC-HRMS screening data.	N1 = 51 blood tests positive for GHB (>10 µg/mL). N2 = 51 blood tests negative for GHB (<10 µg/mL). Various other drugs were found in both groups.	Whole blood	GHB-carnitine was always detected in N1. Succinylcarnitine improves the specificity of GHB-carnitine for the detection of GHB exposure. M259T82, M507T82, and GABA-2-hydroxyglutarate are potential biomarkers of GHB exposure. Using statistical models, the authors distinguished suspected GHB users from non-users with an accuracy of over 80%.
Thimm et al., 2022 [19]	Analytical characterization of phosphatidyl-GHB (P-GHB) and its isomer beta-hydroxybutyric acid (BHB).	Phosphatidyl-GHB and phosphatidyl-BHB were synthetized in vitro, to be then characterized in human whole blood.	Whole blood	P-GHB and P-BHB were successfully synthetized. In vitro, phospholipase D forms P-GHB, making it a potential biomarker for GHB. The study remains very basic. Research in animals and then in humans could be relevant if P-GHB is to become a real candidate. These experiments clearly support our hypothesis that P-GHB is formed by Phospholipase D and might be suitable as a GHB biomarker. For both compounds, the limit of detection established was LOD ≤ 2 ng/mL.
Kim et al., 2022 [20]	Determine the reference concentrations of metabolites that could be used as biomarkers for GHB intoxication.	N = 472 samples from 206 healthy women.	Urine	With advancing age, the creatinine-adjusted concentrations of glutamic acid and succinic acid, as well as succinylcarnitine, significantly increased, whereas the concentration of glycolic acid significantly decreased. The concentrations of GHB, both unadjusted and creatinine-adjusted, were unaffected by differences in age. Significant variation in GABA (LOD = 0.01 µg/mL) concentration was observed between days, as well as significant intra-day variation in the concentrations of 3,4-dihydroxybutyric acid (LOD = 0.21 µg/mL) and succinylcarnitine. The maximum concentration of endogenous GHB detected in urine was 0.75 μg/mL (LOD = 0.17 µg/mL), which is much lower than that previously reported. The strong correlation between the concentrations of 2,4-OH-BA (LOD = 0.24 µg/mL) or 3,4-OH-BA and those of GHB strongly suggests that they could be used as direct markers of GHB intoxication. Although succinic acid (LOD = 0.3 µg/mL) is also correlated, its role in pathological conditions such as inflammation and tumor formation has been emphasized. Therefore, it appears to be unsuitable as a distinct marker for GHB intoxication.
Steuer et al., 2023 [21]	Quantitative characterization of urinary excretion of GHB, GHB-carnitine, GHB-glycine, GHB-glutamate, GHB-taurine, GHB-phenylalanine, GHB-fatty acid esters, and organic acids 2,4-OH-BA, 3,4-OH-BA, GA, SA, and succinylcarnitine after controlled administration of GHB to humans and to evaluate their usefulness for prolonged detection of GHB intake by applying three different discrimination strategies.	Cohort 1 (from 8 years ago): N1 = 10: administration of 42 mg/kg GHB N2 = 10: administration of placebo Urine collected at T4.5h and T8h. Cohort 2 (from 6 month ago): N1 = 40: administration of 42 mg/kg GHB N2 = 40: administration of placebo Urine at T4.5h, T8h, T11h, and T28h.	Urine	GHB-glycine can prolong the detection window of GHB to about 28 h. However, the sensitivity is still lower than that targeted by the authors, which is close to 1. Among all evaluated biomarkers, only GHB-pentose concentrations were below the limit of quantification of 0.05 μg/mL in all placebo samples. GHB fatty acid conjugates could not be detected in any treatment condition. The authors found that endogenous concentrations of GHB and GHB metabolites (placebo) were independent of daytime, with the exception of GHB-glucuronide and succinylcarnitine, which showed significantly lower concentrations in the afternoon. The limit of quantification for all GHB conjugates was 0.05 μg/mL.

## Data Availability

No new data were created or analyzed in this study. Data sharing is not applicable to this article.

## Supplementary Materials

The following supporting information can be downloaded at: https://www.mdpi.com/article/10.3390/toxics13100824/s1, Table S1: Summary of the sample preparation and analytical techniques used in each of the studies included in the review.

## Abbreviations

The following abbreviations are used in this manuscript:

2,4-OH-BA	2,4-dihydroxybutyric acid
2-HG	2-hydroxyglutaric acid
3,4-OH-BA	3,4-dihydroxybutyric acid
4 HPA	4-hydroxyphenylacetic acid
ACN	Acetonitrile
AA	Adipic acid
BHB	Beta-hydroxybutyric acid
BSTFA	N,O-bis(triméthylsilyl)trifluoroacétamide
DFSA	Drug-facilitated Sexual Assaults
GABA	Gamma-aminobutyric acid
GBA	4-Guanidinobutyric acid
GHB	Gamma-hydroxybutyric
GHB-GLU	GHB-glucuronide
GHB-SUL	GHB sulfate
GA	Glycolic acid
GC-MS	Gas chromatography–mass spectrometry
LOQ	Limit of quantification
LLE	Liquid–liquid extraction
LC-HRMS	Liquid chromatography–high-resolution mass spectrometry
LC-MS/MS	Liquid chromatography–tandem mass spectrometry
N	Populations studied
PAs	Urinary polyamines
P-GHB	Phosphatidyl-GHB
SA	Succinic acid
TCA	Tricarboxylic acid
UHPLC-HRMS	Ultra-high-performance liquid chromatography high-resolution–high-resolution mass spectrometry
