# Supplementary material for: Biomarkers of Gamma-Hydroxybutyric Acid (GHB) Exposure: A Comprehensive Review of Analytical and Forensic Advances
Source: Toxics. 2025 Sep 27;13(10):824. doi: 10.3390/toxics13100824 (PMC12567942; doi:10.3390/toxics13100824)
Supplement: Supplementary file 1 [file toxics-13-00824-s001.zip › toxics-3816758-supplementary.pdf]

Table S1. Summary of the sample preparation and analytical techniques used in each of the studies included in the review.

Reference	Analytical Technique	Sample Preparation
Animal study (Sprague-Dawley rats)		
Seo et al.<br>2016<br>[7]	Chromatographic column:<br>Ultra-2 (5% phenyl - 95% methylpolysiloxane; 25 m x 0.2 mm I.D. 0.11 µm)<br><br>Ionization source:<br>Electron impact (EI)<br><br>GC-MS:<br>GC: 7890N (Agilent)<br>MS: 5975C (Agilent)	Sample volume : not specified<br><br>Extraction: Liquid-liquid extraction (LLE), diethyl ether (3 mL) and ethyl acetate (2 mL)<br>Derivatization with toluene: MTBSTFA* (50:50;v:v).<br>**N-tert-butyldiméthylsilyl-N-méthyltrifluoroacétamide*
Seo et al.<br>2018 [8]	Chromatographic column:<br>Ultra-2 (5% phenyl - 95% methylpolysiloxane; 25 m x 0.2 mm I.D. 0.11 µm)<br><br>Ionization source:<br>Electron impact (EI)<br><br>GC-MS:<br>GC: 7890N (Agilent)<br>MS: 5975C (Agilent)	Sample volume : not specified<br><br>Extraction: LLE, diethyl ether (3 mL) and ethyl acetate (2 mL)<br>Derivatization with toluene: MTBSTFA* (50:50;v:v).<br>**N-tert-butyldiméthylsilyl-N-méthyltrifluoroacétamide*
Lee et al.<br>2019 [9]	Chromatographic column:<br>Ultra-2 (5% phenyl - 95% methylpolysiloxane; 25 m x 0.2 mm I.D. 0.11 µm)<br><br>Ionization source:<br>Electron impact (EI)<br><br>GC-MS: TQ8040 (Shimadzu)	Samples volume: urine (equivalent to ca. 0.1 mg of creatinine) and internal standard were spiked to an aliquot of distilled water (ca. 1 mL) and adjusted to pH ≥ 12 with 5.0 M NaOH.<br><br>Extraction: two-phase extractive N-ethoxycarbonyl-N-pentafluoropropionyl (EOC/PFP) reaction by vortex-mixing with ethyl chloroformate (ECF) in a dichloromethane phase. The mixture was saturated with NaCl and extracted sequentially with diethyl ether, ethyl acetate, and a mixture of diethyl ether and dichloromethane The combined extracts were evaporated to dryness, derivatized with pentafluoropropionyl anhydride (PFPA), evaporated again, and finally dissolved in toluene.
Kim et al.<br>2025 [6]	Chromatographic column :<br>Acquity UPLC HSS T3 (Waters) (2.1 mm × 100 mm, 1.8 µm)<br><br>Ionization mode:	Sample volume : 20 µL<br>Centrifuged and filtered urine samples were mixed with a working internal standard solution, sodium carbonate, and

	ESI +<br>ESI -<br><br>LC-MS:<br>1290 infinity LC system (Agilent) coupled with a 6530 Q-TOF (Agilent)	2% BzCl in acetonitrile (ACN) for derivatization.
Human study		
Petersen et al. 2013 [10]	Chromatographic column: XDB-C18 (50 x 4.6 mm, 1.8 µm, Agilent Technologies)<br><br>LC-MS/MS :<br>6460 triple-quadrupole (Agilent Technologies)	Sample volume: 250 µL<br><br>Extraction method : Solid phase extraction (SPE) OASIS MCX (60 mg/3cc Waters)<br><br>Ionization mode:<br>ESI+
Hanish et al. 2015 [11]	Chromatographic column: Luna C18 (150 x 2 mm, 5 µm, Phenomenex)<br><br>LC-MS/MS :<br>API 4000 triple-quadrupole (Sciex)	Sample volume : 100 µL<br><br>Extraction method : LLE with 200 µL ACN.<br><br>Ionization mode:<br>ESI+
Piper et al. 2017 [12]	Chromatographic column:<br>HYPERCARB (Thermo Scientific)<br><br>LC-MS/MS:<br>LC 1290 Infinity HPLC (Agilent)<br>G6550A Q-TOF (Agilent)	Sample volume: 100 µL<br><br>Extraction method :<br>No extraction<br><br>Ionization source:<br>ESI-
Palomino-Schätzlein et al. 2017 [13]	NMR:<br>Bruker AVANCE II 600	Sample volume: 1.8 mL<br><br>Extraction: 1.8 mL are lyophilized (serum and urine), reconstituted with 0.45 mL for serum and 0.9 mL for urine of deuterated water (D_2O), then diluted in 200 µL of sodium phosphate buffer solution, transferred directly to the 5 mm tube for NMR analysis.
Steuer et al. 2019 [14]	Chromatographic column :<br>- XSelect HSST RP-C18 column (150 mm x 2.1 mm, particle size 2.5 µm) (Waters)<br>- HILIC column (150 mm x 2.1 mm, particle size 3.5 µm) (Merck)<br><br>Ionization mode:<br>ESI +<br>ESI -<br><br>LC-HRMS:	Sample volume: 200 µL<br><br>Extraction method: dilution with 200 µL mobile phase.

	Ultimate 3000 (ThermoFisher) Q-TOF (Triple TOF 6600, Sciex)	
Steuer et al.<br>2021 [15]	Chromatographic column :<br>- XSelect HSST RP-C18 column (150 mm x 2.1 mm, particle size, 2.5 µm) (Waters)<br>- HILIC column (150 mm x 2.1 mm, particle size 3.5 µm) (Merck)<br><br>Ionization mode:<br>ESI +<br>ESI –<br><br>LC-HRMS:<br>Ultimate 3000 (ThermoFisher) Q-TOF (Triple TOF 6600, Sciex)	Sample volume :<br>75 µL for serum<br>200 µL for urine<br><br>Extraction method: dilution with 200 µL mobile phase.
Jarsiah et al.<br>2021 [16]	Chromatographic column:<br>TG-5SILMS (30 m x 0.25 mm ID, 0.25 µm) (Agilent technology).<br><br>Ionization source:<br>Electron impact (EI)<br><br>GC-MS:<br>GC: 7890A (Agilent) MS: MSD 5975C (Agilent)	Sample volume: 1 mL serum or urine.<br><br>Extraction method: LLE (5 mL ethyl acetate).
Küting et al.<br>2021 [17]	Chromatographic column:<br>HP-5MS (Agilent)<br><br>Ionization source:<br>Electron impact (EI)<br><br>GC-MS:<br>GC: 6890N GC (Agilent)<br>MS: 5973N (Agilent)	Sample volume:<br>50 µL of plasma or urine.<br><br>Extraction method: samples were mixed with 140 µL.<br>Supernatant was evaporated to dryness before derivatization with 50 µL of BSTFA (with 1% TMCS) and 100 µL of isooctane.
Wang et al.<br>2022 [18]	Chromatographic column:<br>ACQUITY BEH C18 (100 mm x 2.1 mm, 1.7 µm) (Waters)<br><br>Ionization mode:<br>ESI +<br><br>UHPLC-HRMS:<br>ACQUITY I-Class (Water)	Sample volume: 300 µL<br><br>Extraction method: precipitation with a mixture of methanol (200 µL) and acetonitrile (300 µL).
Thimm et al.<br>2022 [19]	Chromatographic column:<br>Reprosil-Pur ODS-3 (2 x 500 mm, 3 µm)(Altmann Analytik)<br><br>LC-HRMS	Sample volume: 150 µL heparinized blood.<br><br>Extraction method:<br>LLE with 750 µL methanol/ethyl acetate (1:1, v/v).

	Agilent 1200 (Agilent)<br>Bruker microTOF-Q II (Bruker)<br><br>NMR<br>Varian INOVA-500	
Kim et al.<br>2022 [20]	Chromatographic column :<br>Acquity UPLC HSS T3 (Waters) (2.1 mm × 100 mm, 1.8 µm)<br><br>Ionization mode:<br>ESI +<br>ESI -<br><br>LC-MS:<br>1290 infinity LC system (Agilent) 6495 triple quadrupole MS/MS (Agilent)	Sample volume:<br>20 µL of urine.<br><br>Extraction method: urine samples were mixed with 10 µL of 100 mM sodium carbonate buffer and benzoyl chloride (2% (v/v) in acetonitrile) for derivatization.
Steuer et al.<br>2023 [21]	Chromatographic column :<br>SeQuant ZIC-HILIC column (Merck) (150 × 2.1 mm, 3.5-µm)<br><br>Ionization mode:<br>ESI +<br>ESI -<br><br>LC-HRMS:<br>LC-40Dx3 LC system (Shimadzu)<br>5500 QTtrap (Sciex)	Sample volume:<br>100 µL of urine<br><br>Extraction method: dilution with 500 µL of acetonitrile.
